# Supplementary material for: AFsample2 predicts multiple conformations and ensembles with AlphaFold2
Source: Commun Biol. 2025 Mar 5;8:373. doi: 10.1038/s42003-025-07791-9 (PMC11882827; doi:10.1038/s42003-025-07791-9)
Supplement: Supplementary file 1 — Supplementary Information [file 42003_2025_7791_MOESM1_ESM.pdf]

Supplementary Information: AFsample2 predicts multiple conformations and ensembles with AlphaFold2

Yogesh Kalakoti[a] **and Björn Wallner**[a] ✉

[a]Division of Bioinformatics, Department of Physics, Chemistry and Biology, Linköping University, 581 83 Linköping, Sweden

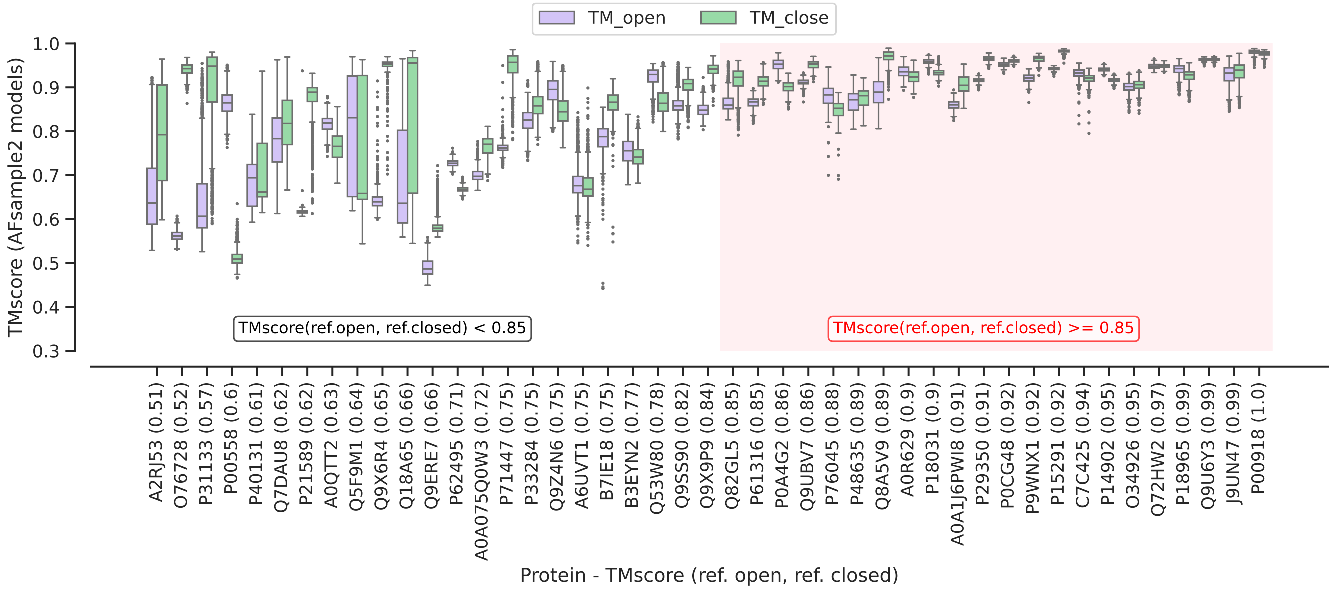

Fig. S1. Distribution of TM-scores for all models generated by AFsample2 for OC23 and OC>85 (shaded region) datasets.

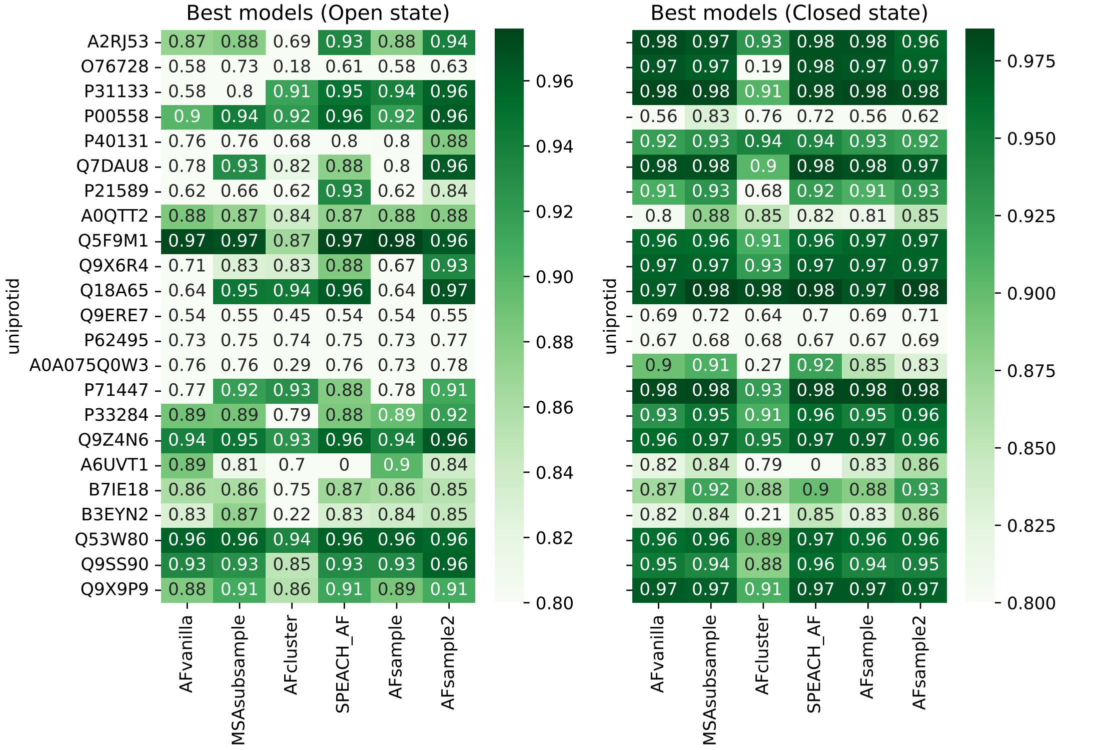

(a)

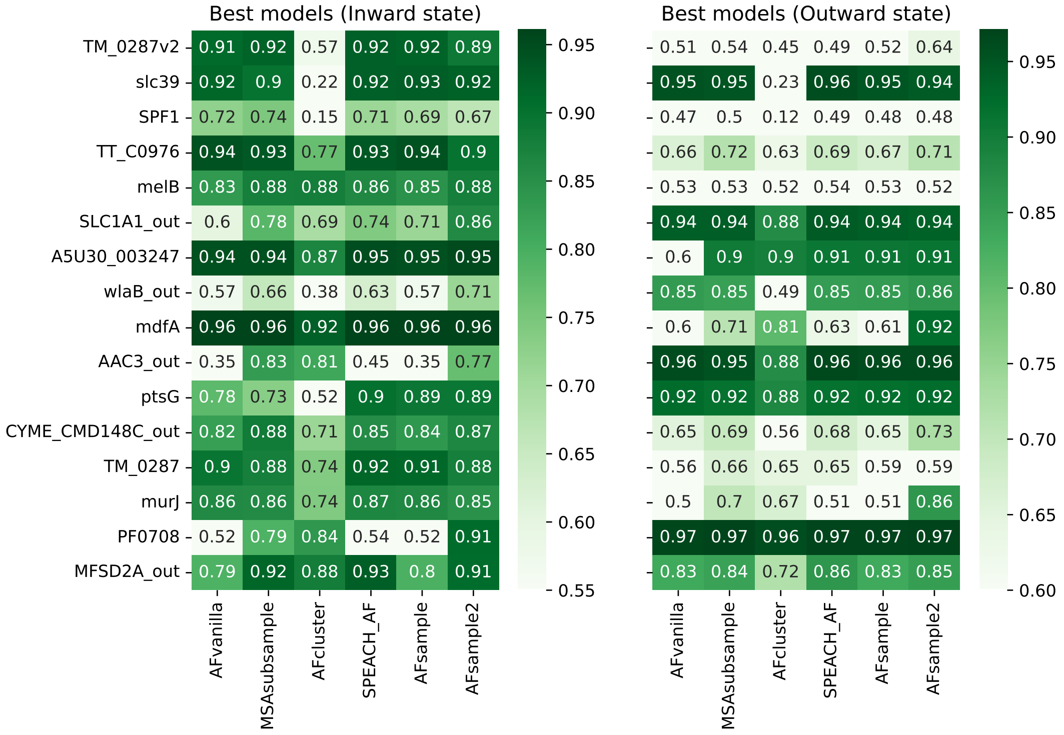

(b)

Fig. S2. Best models generated by all competing methods for the (a) OC23 and (b) TP16 dataset.

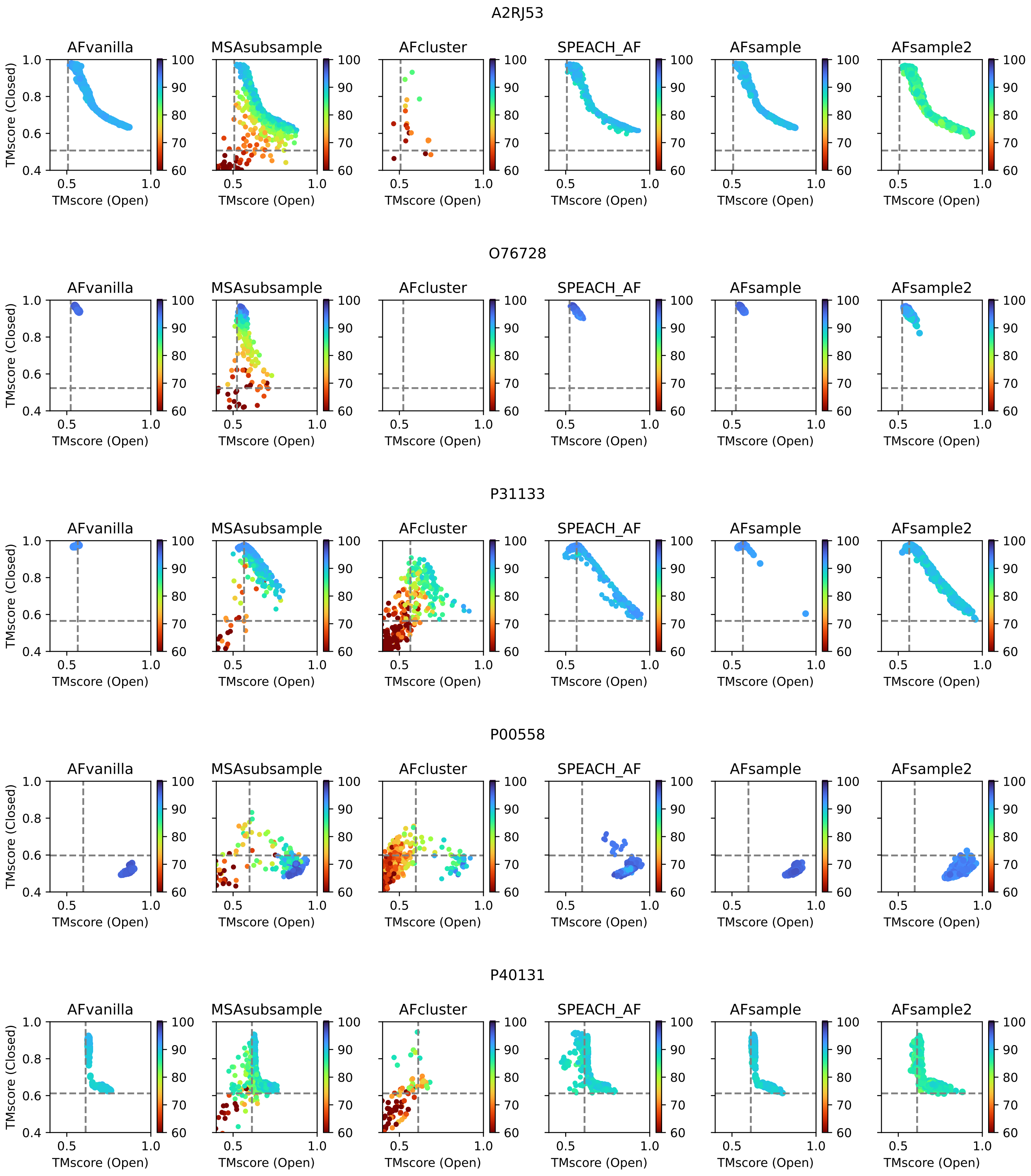

Fig. S3. Diversity plots to capture the similarity of model ensembles with reference states for all targets in the OC23 dataset (continued on next page)

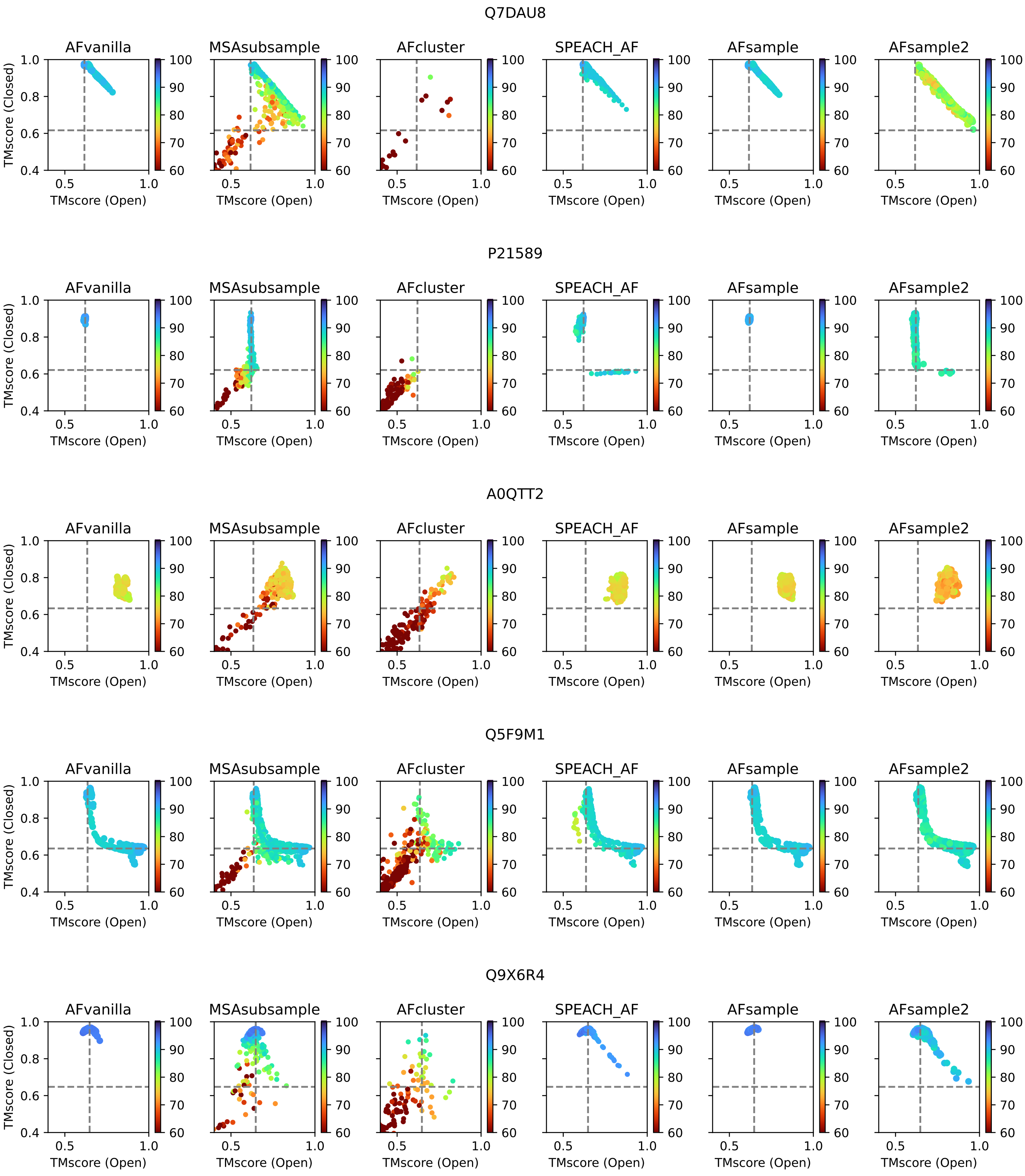

Fig. S3. Diversity plots to capture the similarity of model ensembles with reference states for all targets in the OC23 dataset (continued on next page)

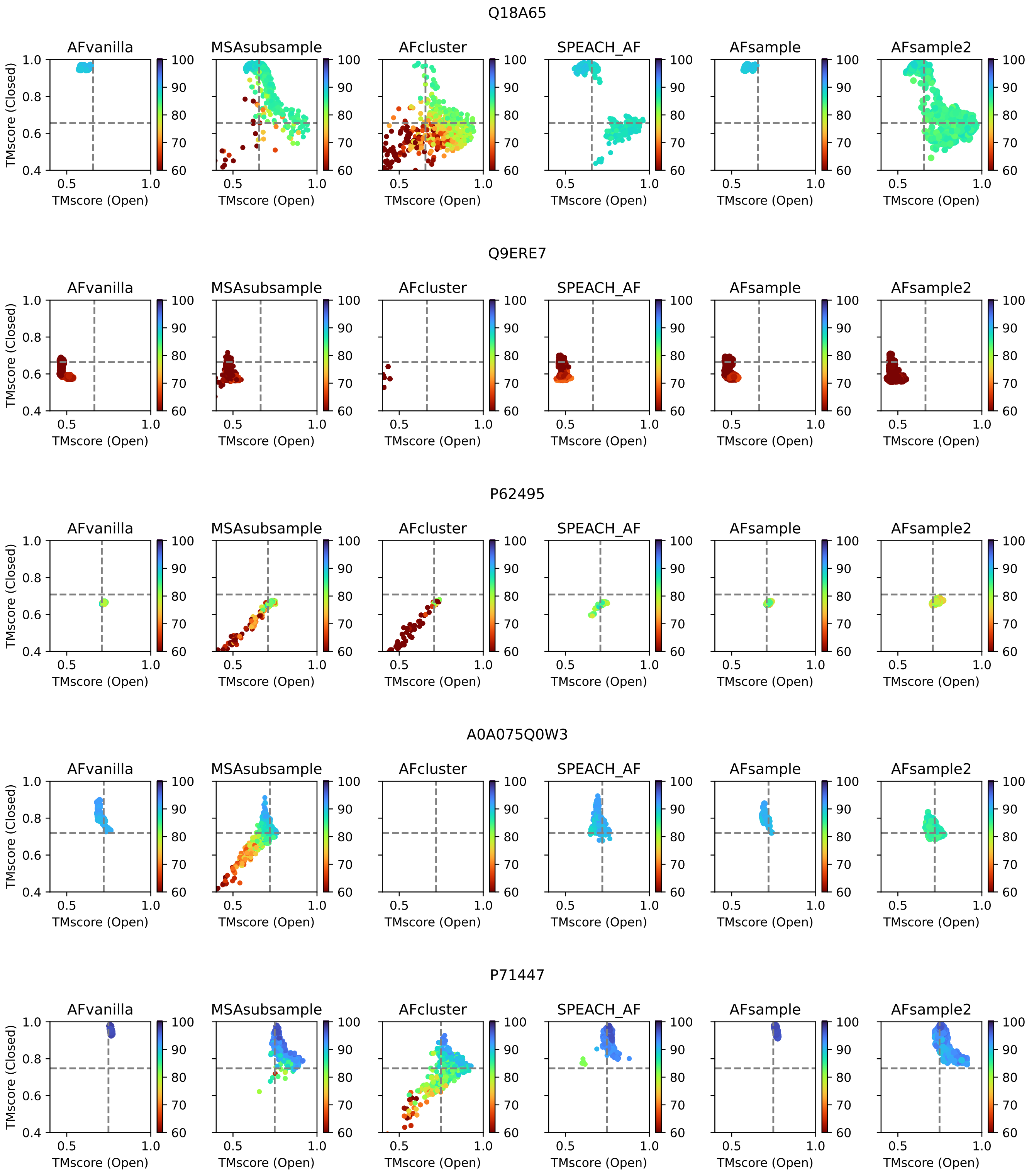

Fig. S3. Diversity plots to capture the similarity of model ensembles with reference states for all targets in the OC23 dataset (continued on next page)

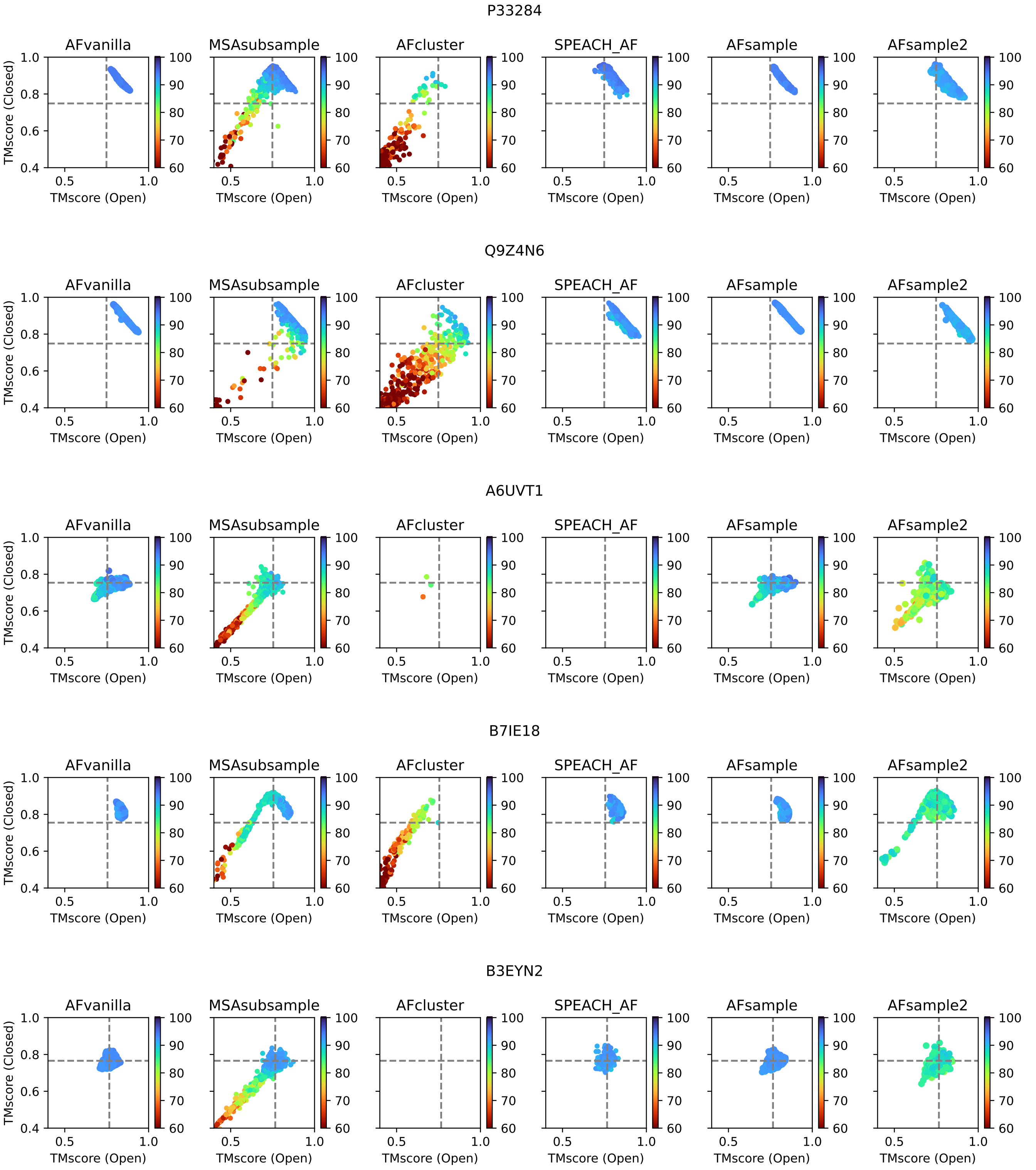

Fig. S3. Diversity plots to capture the similarity of model ensembles with reference states for all targets in the OC23 dataset
(continued on next page)

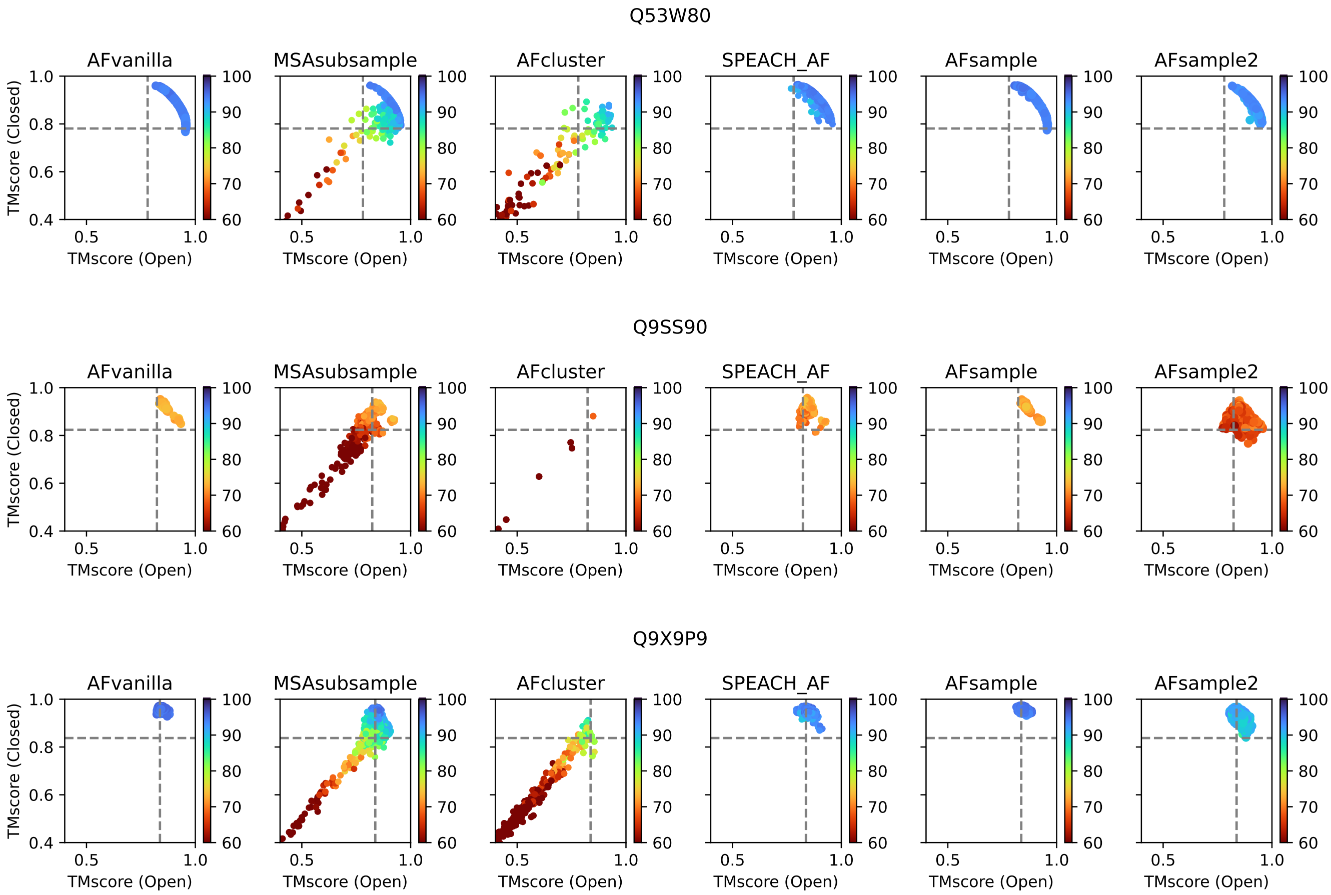

Fig. S3. Diversity plots to capture the similarity of model ensembles with reference states for all targets in the OC23 dataset

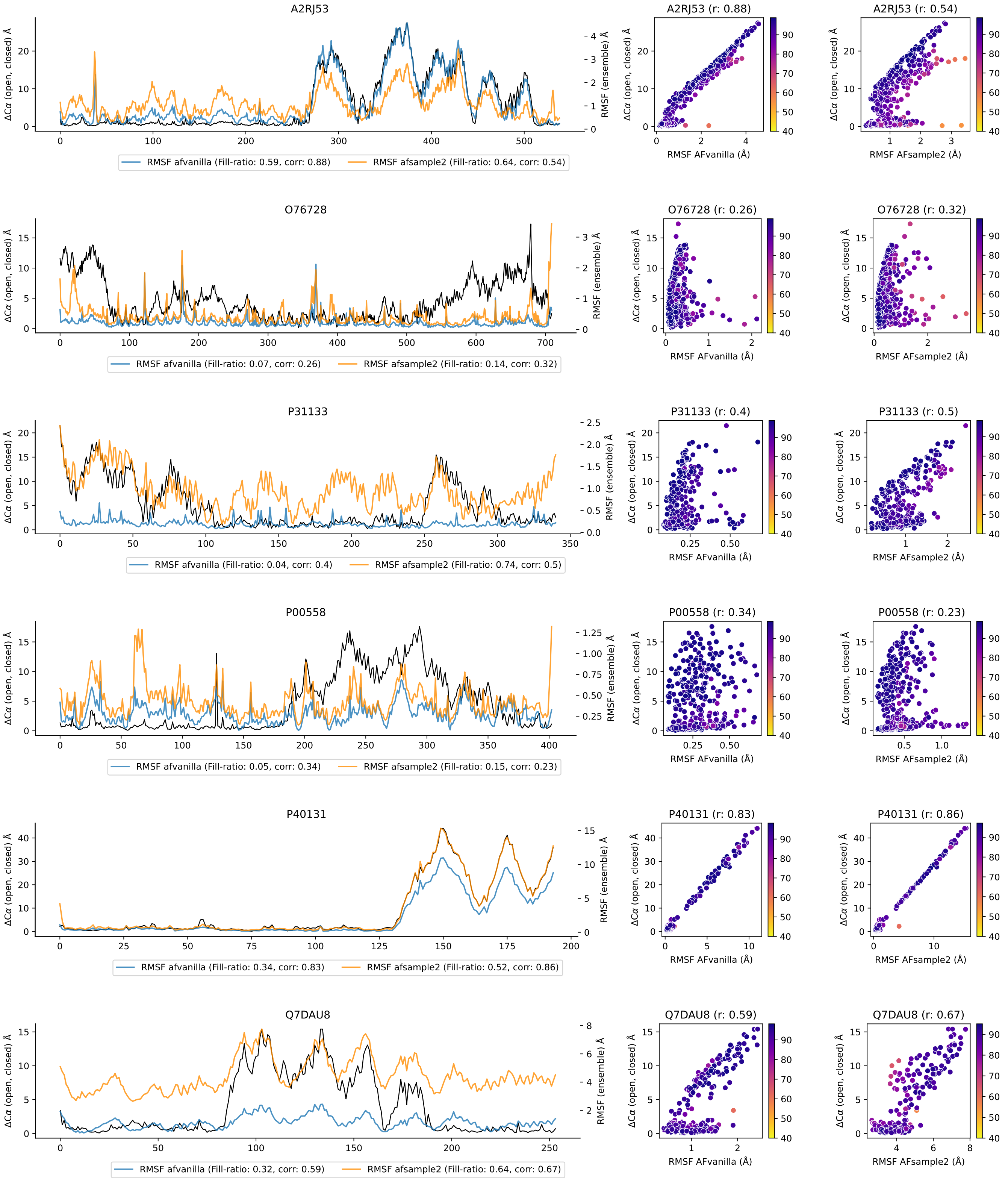

Fig. S4. Correlation between fluctuations observed in the generated model ensemble and reference states at a residue level (continued on next page)

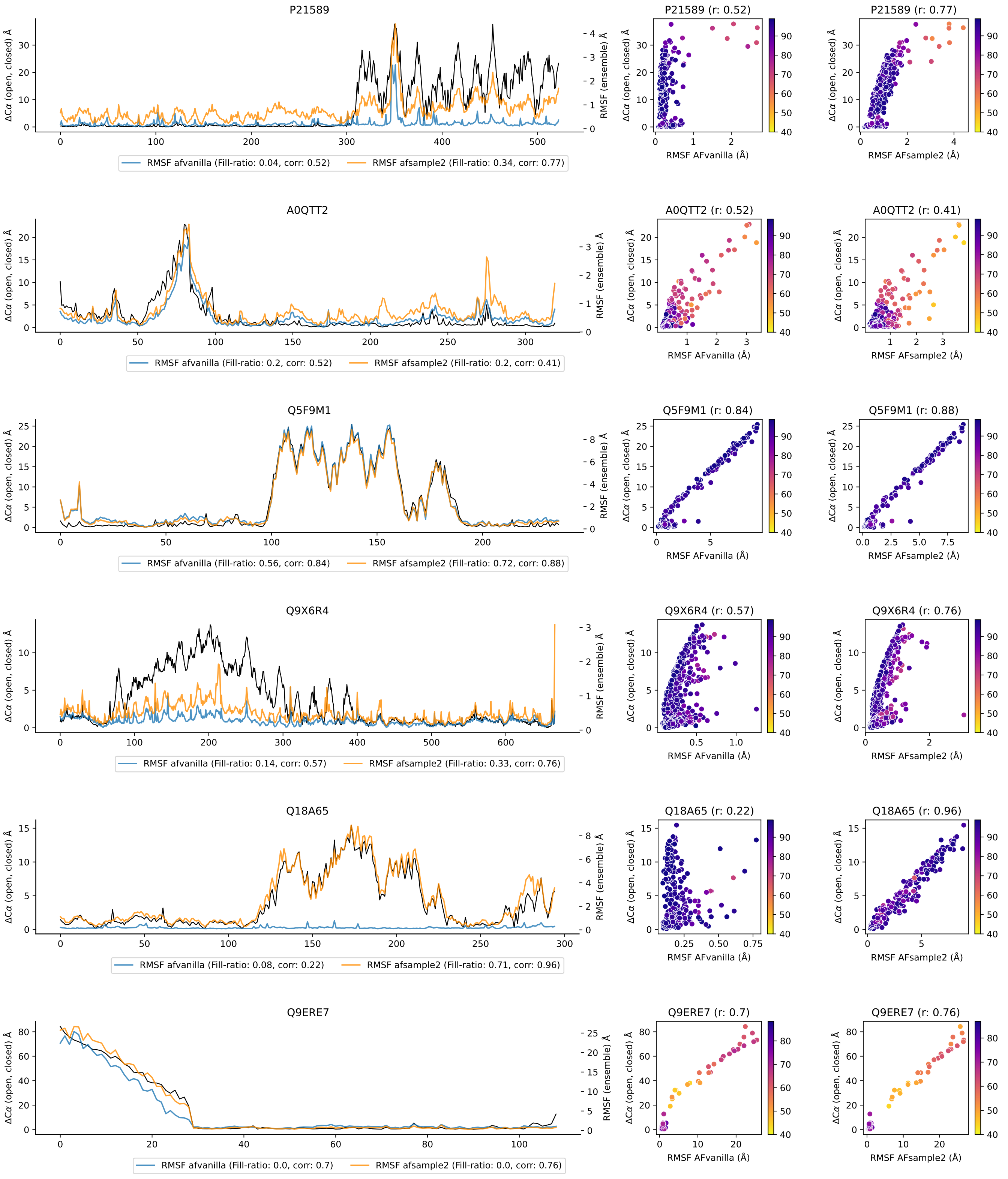

Fig. S4. Correlation between fluctuations observed in the generated model ensemble and reference states at a residue level
(continued on next page)

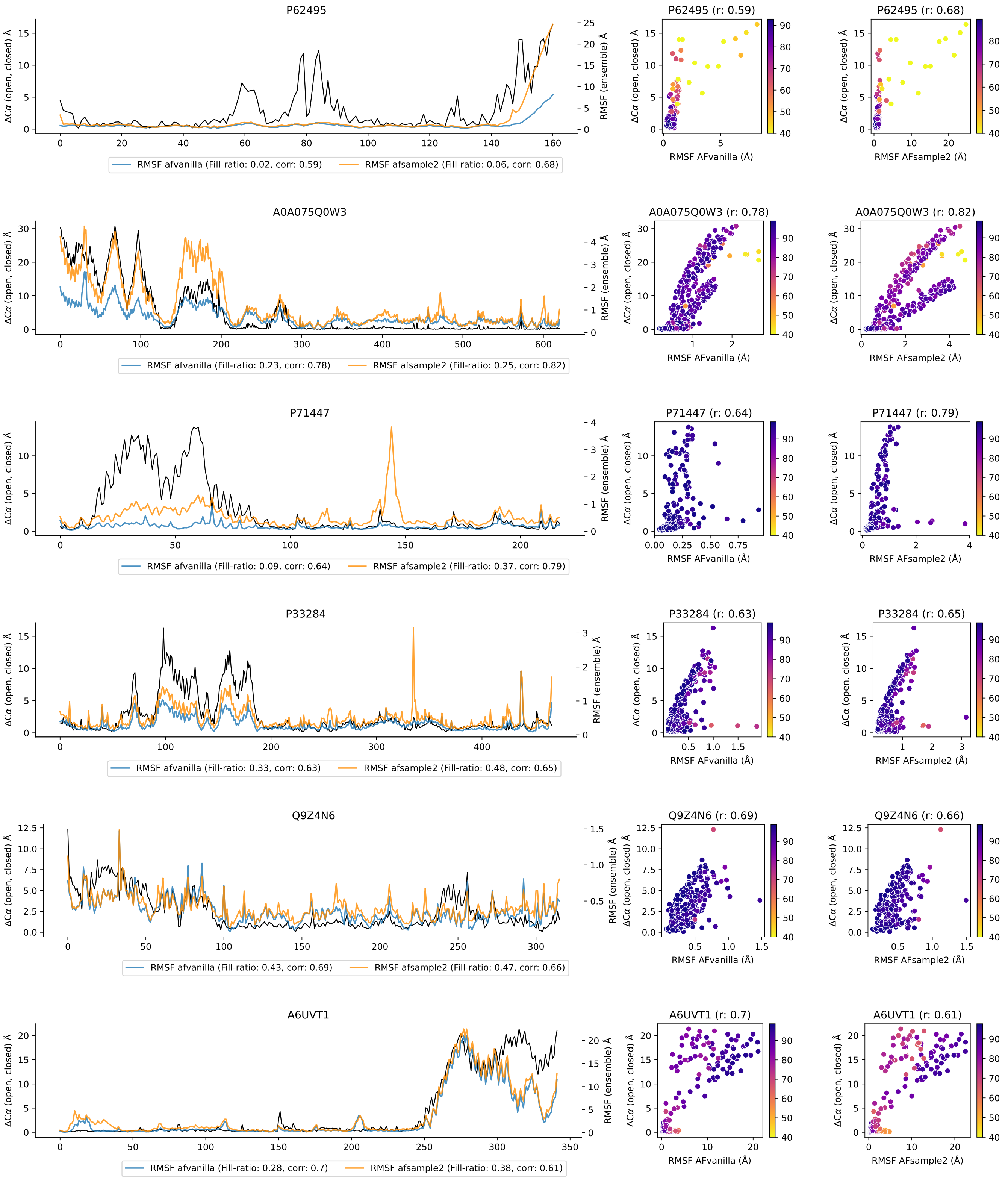

Fig. S4. Correlation between fluctuations observed in the generated model ensemble and reference states at a residue level (continued on next page)

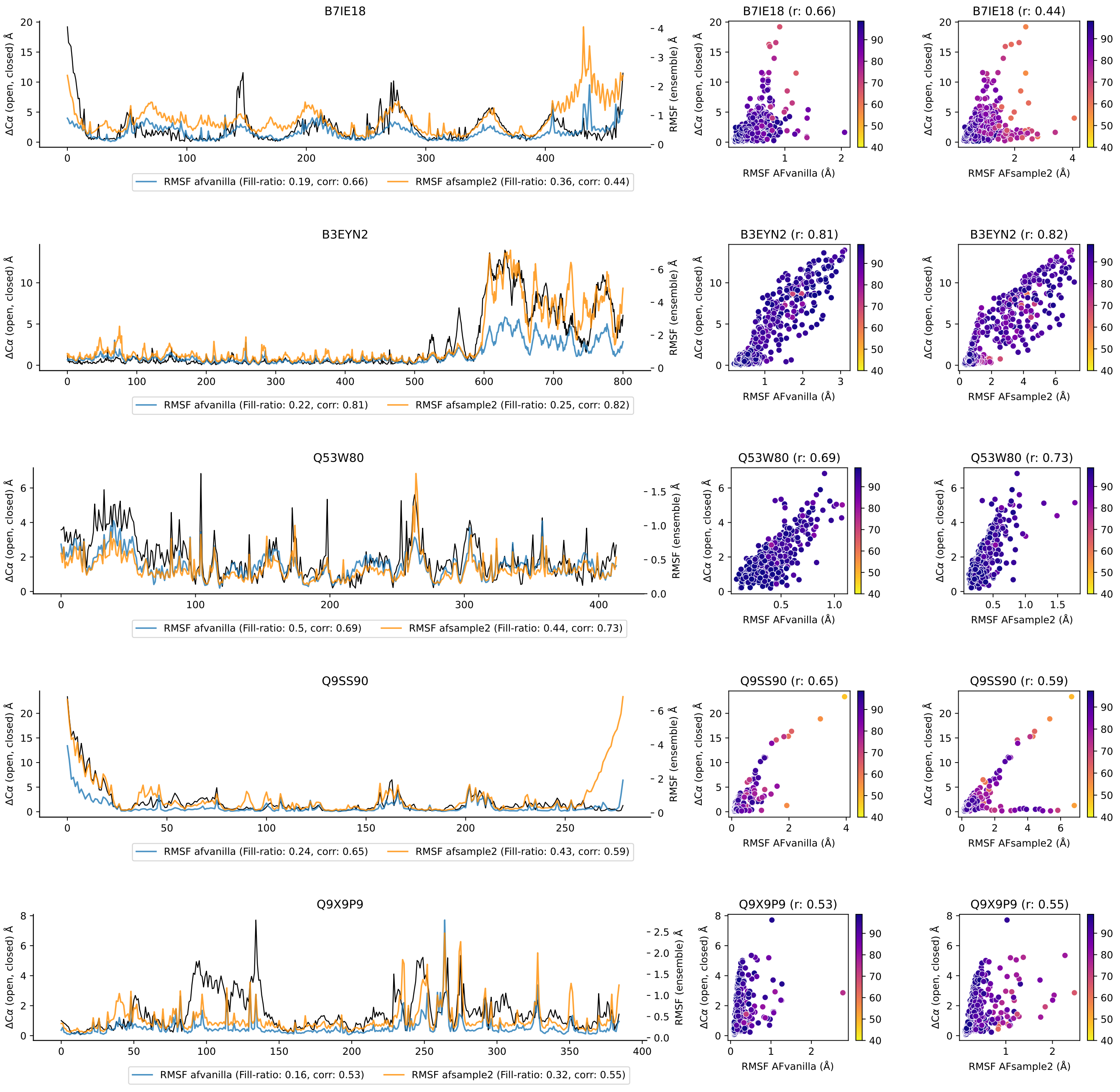

Fig. S4. Correlation between fluctuations observed in the generated model ensemble and reference states at a residue level

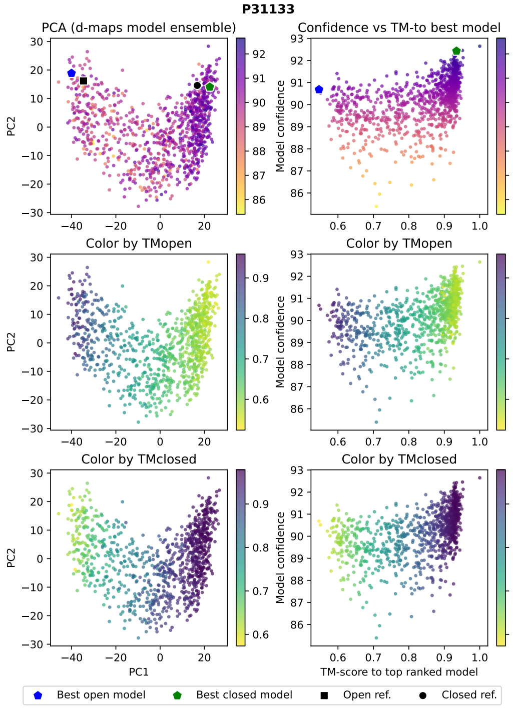

(a) PC1 separation

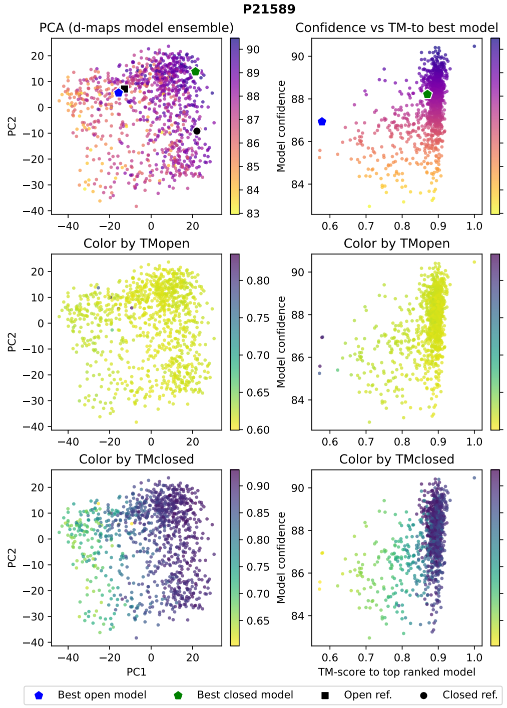

(b) Few good open state models

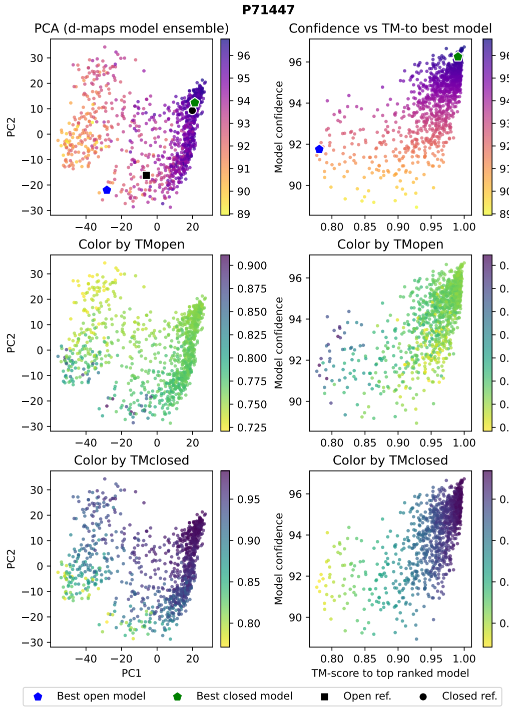

(c) No separation in PC1/PC2

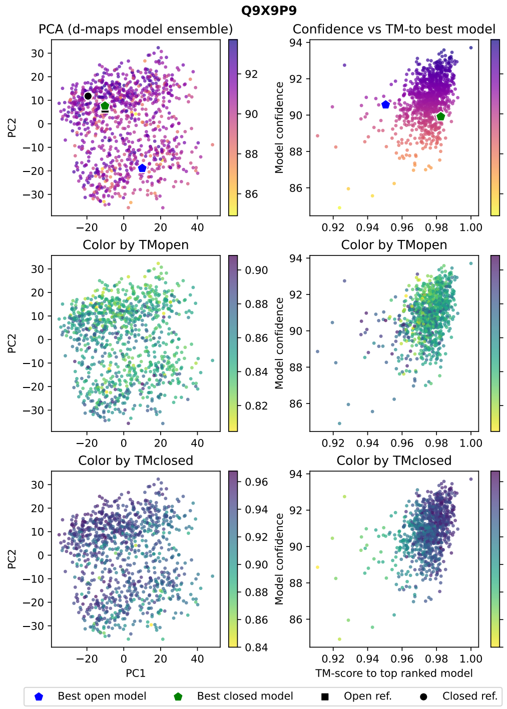

(d) No separation in PC1/PC2

Fig. S5. PCA and TMrank1 vs model confidence. For different targets PCA (left) TMrank1 (right), colored from the top using model confidence, TM to open, and TM to closed, respectively. While PCA, in many cases, is able to generate a relevant separation of the ensemble, it is not obvious how to select states. In (a), there is a separation in PC1 space, but it is not obvious why models with high PC2 should be picked, in contrast, in TMrank1/confidence space you would pick high-confident models structurally different from rank1 model, (b) Few good models for the open state no separation in PC1/PC2 space, but good separation in TMrank1/confidence space, (c) No separation in PC1/PC2 space, (d) No separation in PC1/PC2 space.

(continued on next page)

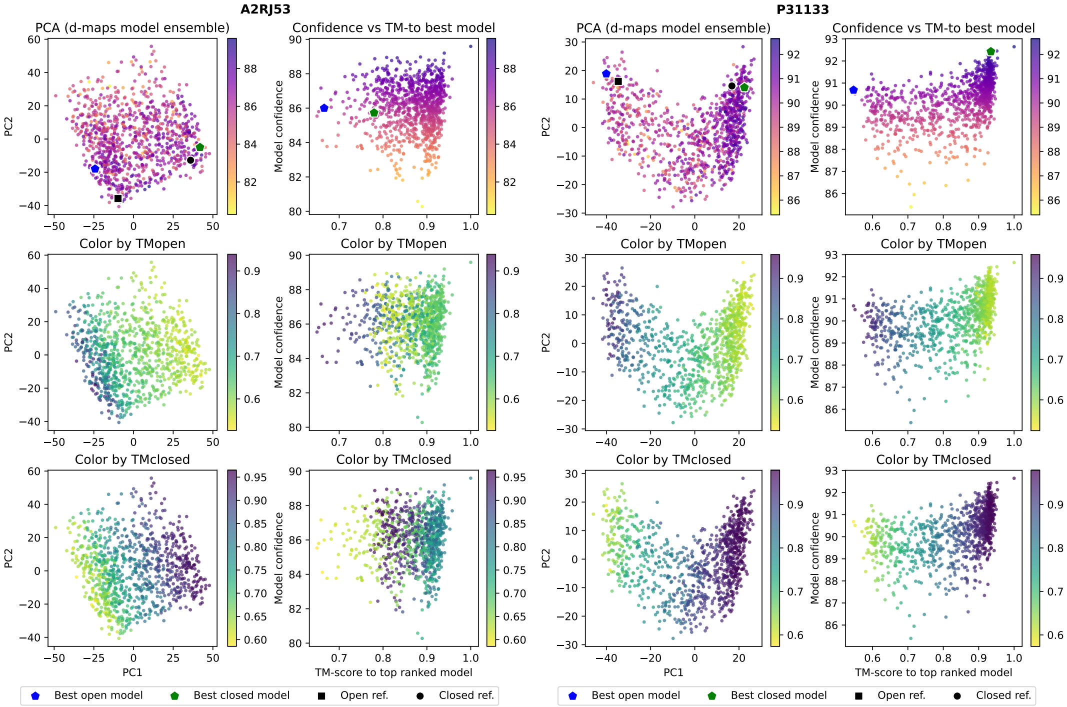

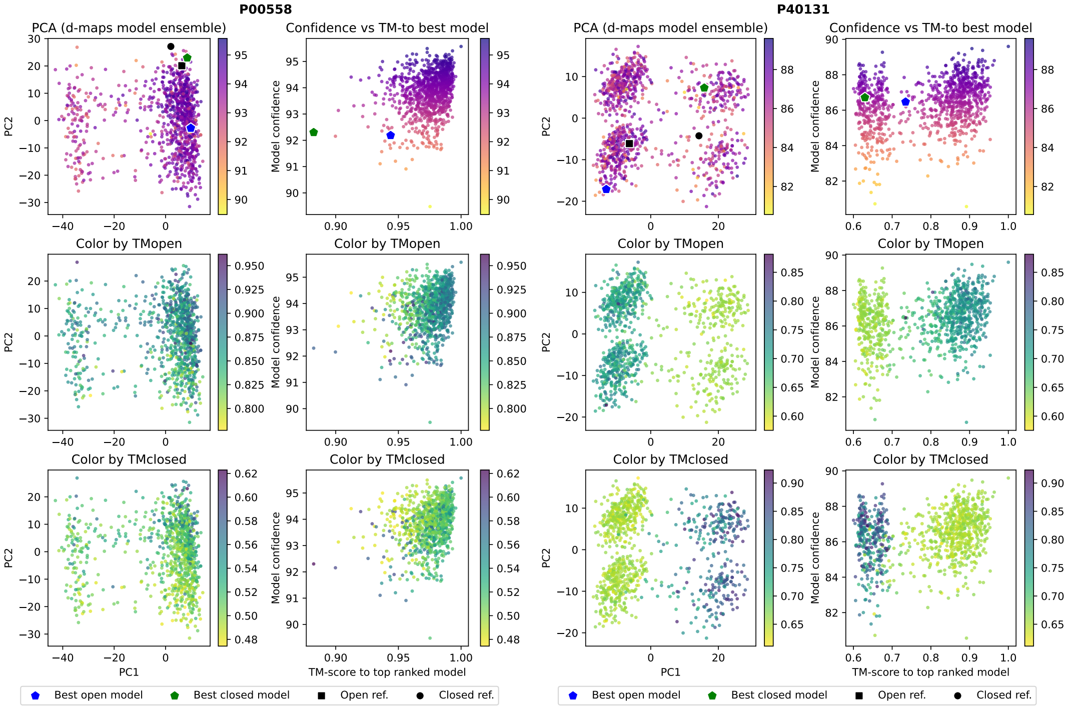

Fig. S6. PCA and TMrank1 vs model confidence. For different targets PCA (left) TMrank1 (right), colored from the top using model confidence, TM to open, and TM to closed, respectively. (continued on next page)

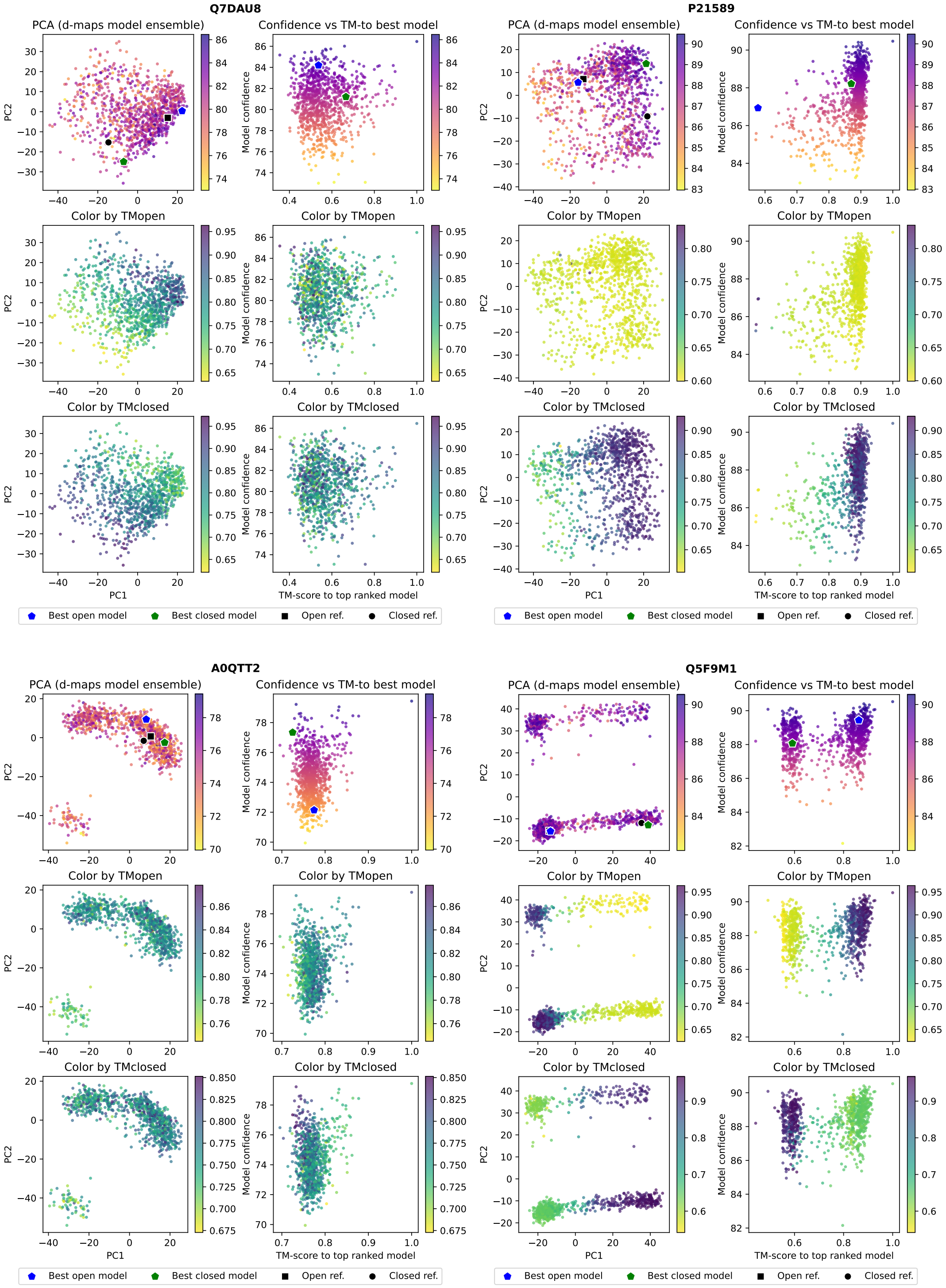

Fig. S6. PCA and TMrank1 vs model confidence. For different targets PCA (left) TMrank1 (right), colored from the top using model confidence, TM to open, and TM to closed, respectively.(continued on next page)

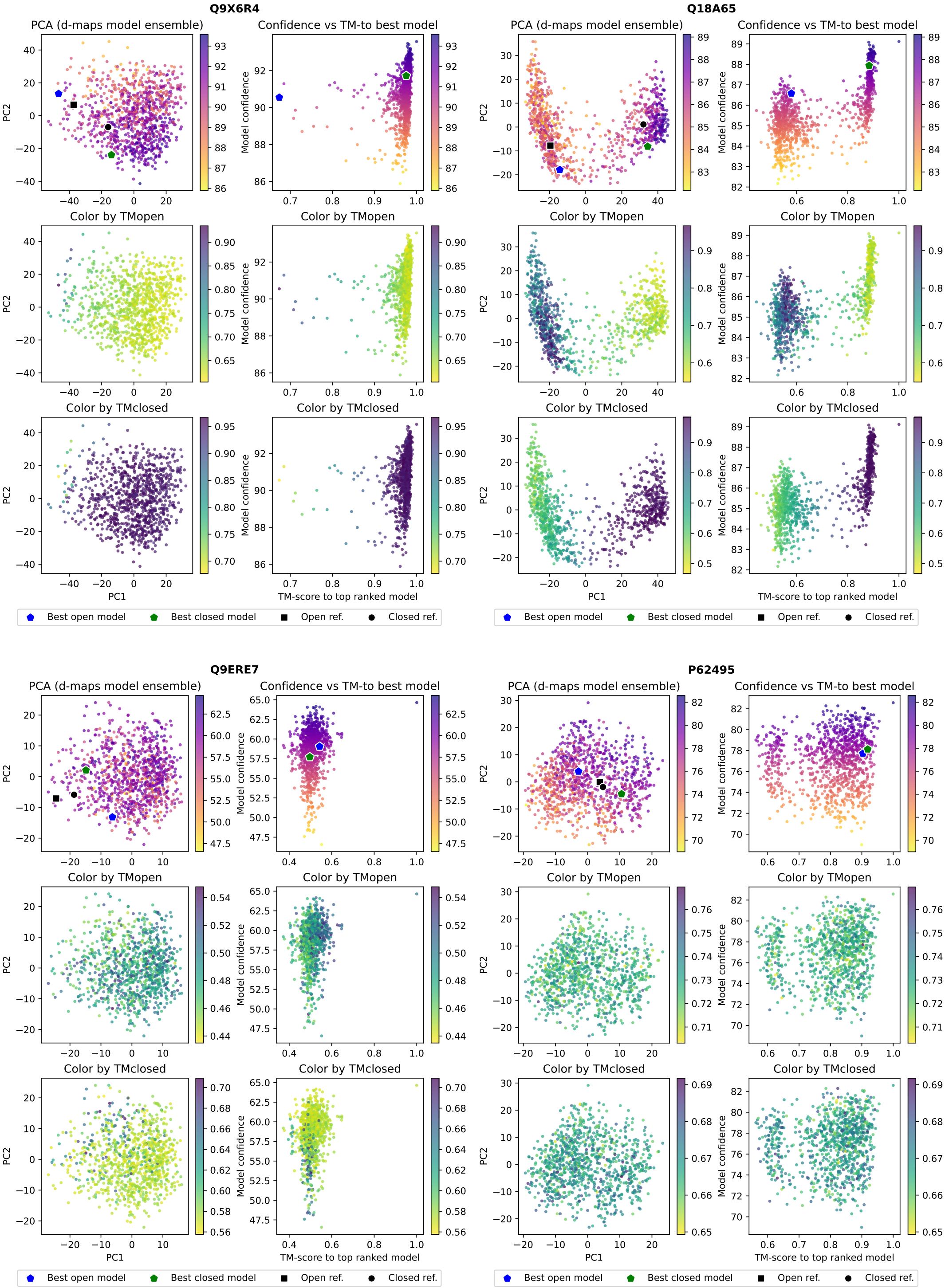

Fig. S6. PCA and TMrank1 vs model confidence. For different targets PCA (left) TMrank1 (right), colored from the top using model confidence, TM to open, and TM to closed, respectively.(continued on next page)

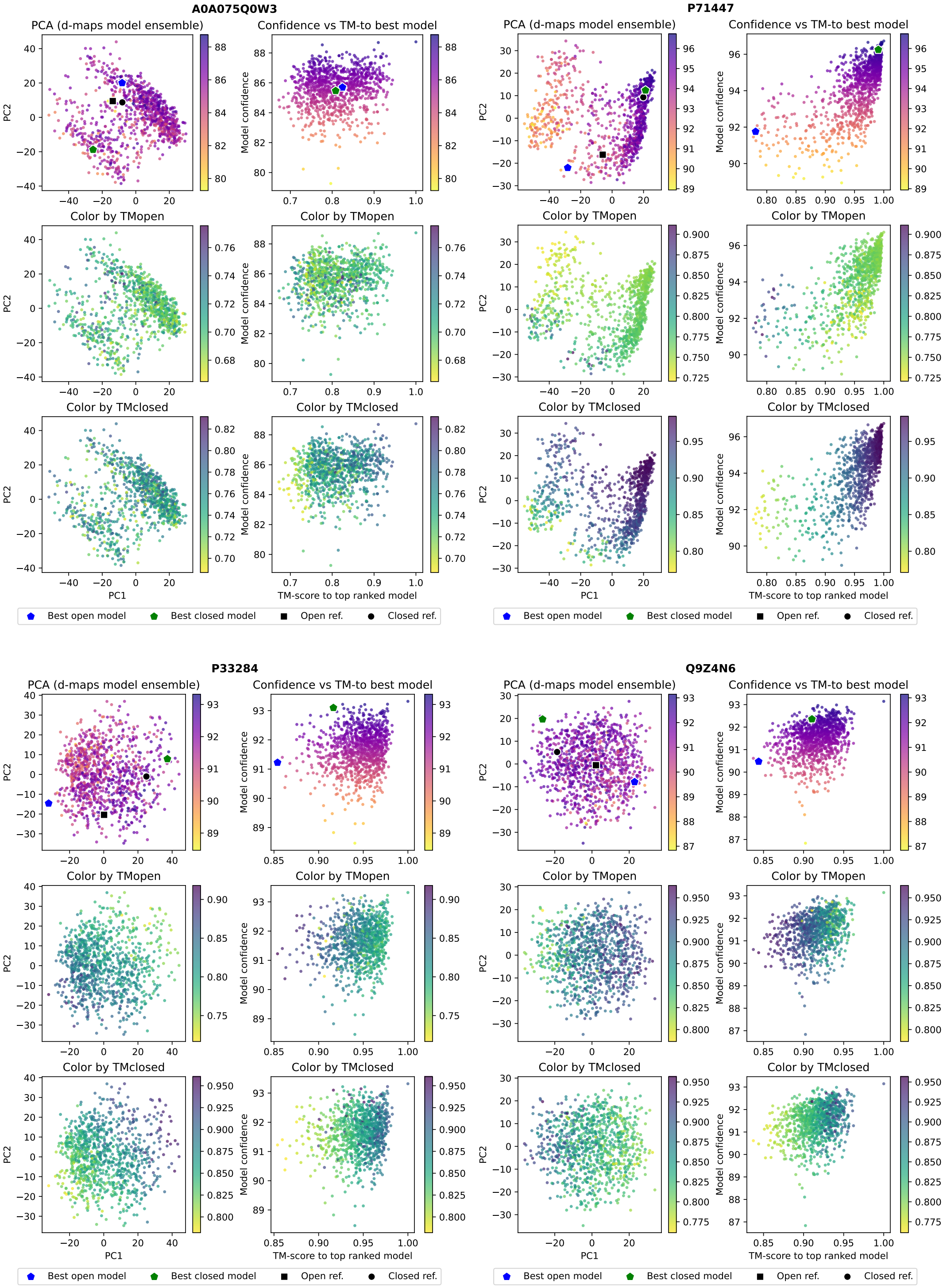

Fig. S6. PCA and TMrank1 vs model confidence. For different targets PCA (left) TMrank1 (right), colored from the top using model confidence, TM to open, and TM to closed, respectively.(continued on next page)

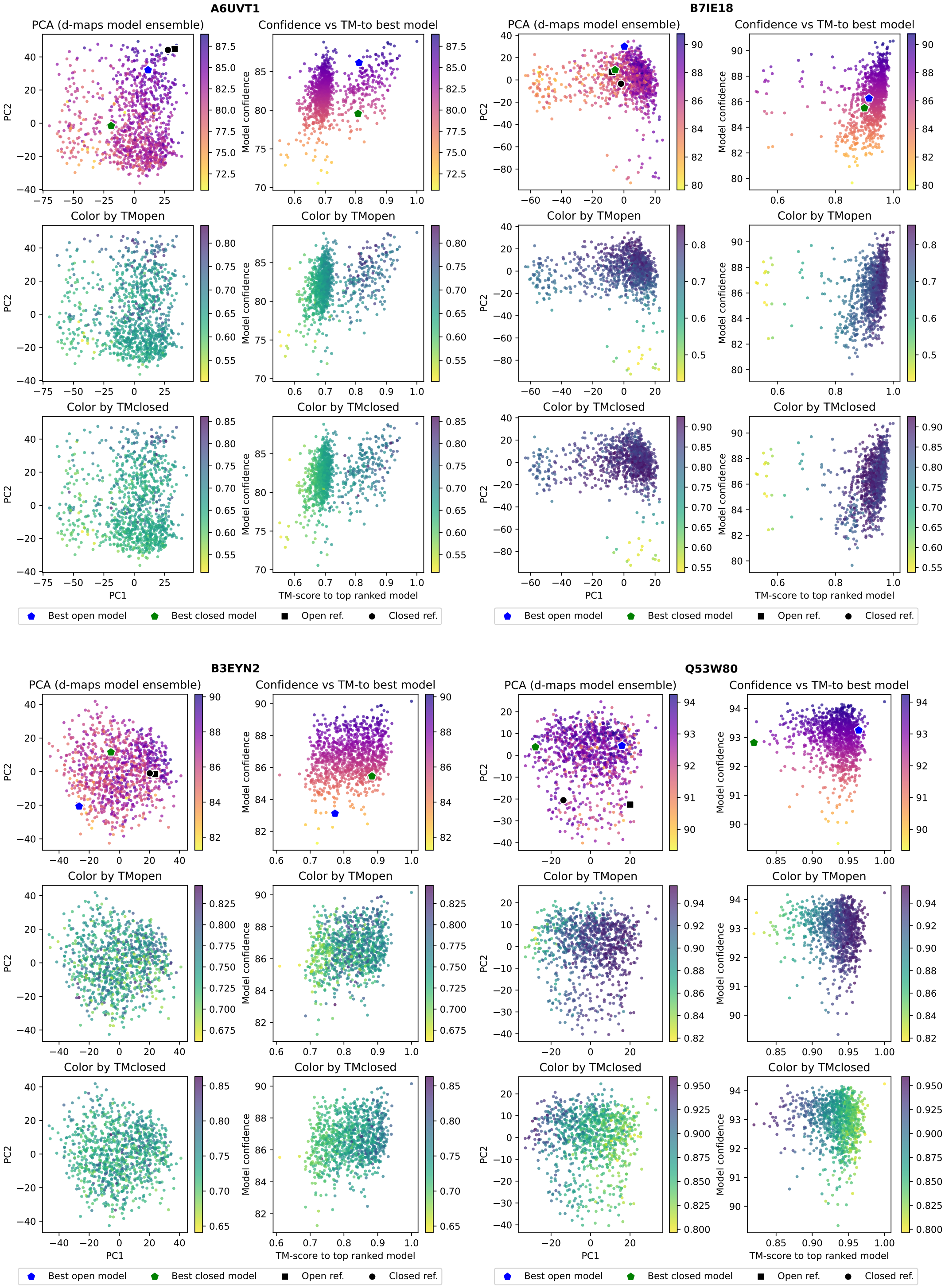

Fig. S6. PCA and TMrank1 vs model confidence. For different targets PCA (left) TMrank1 (right), colored from the top using model confidence, TM to open, and TM to closed, respectively.(continued on next page)

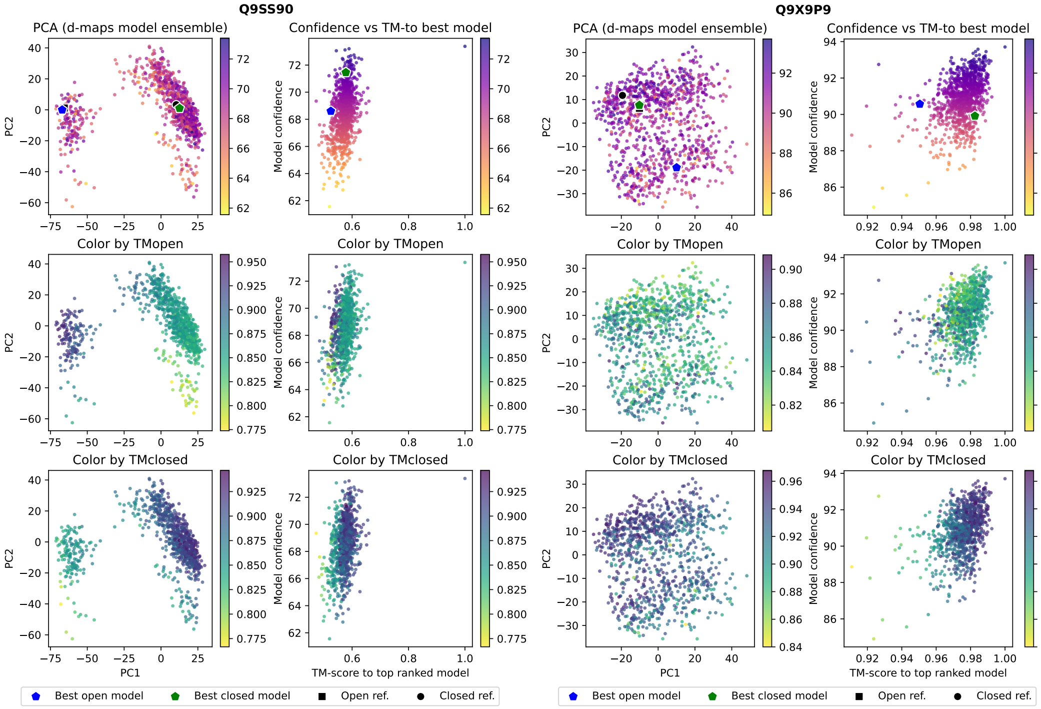

Fig. S6. PCA and TMrank1 vs model confidence. For different targets PCA (left) TMrank1 (right), colored from the top using model confidence, TM to open, and TM to closed, respectively.(do not continue on next page...)

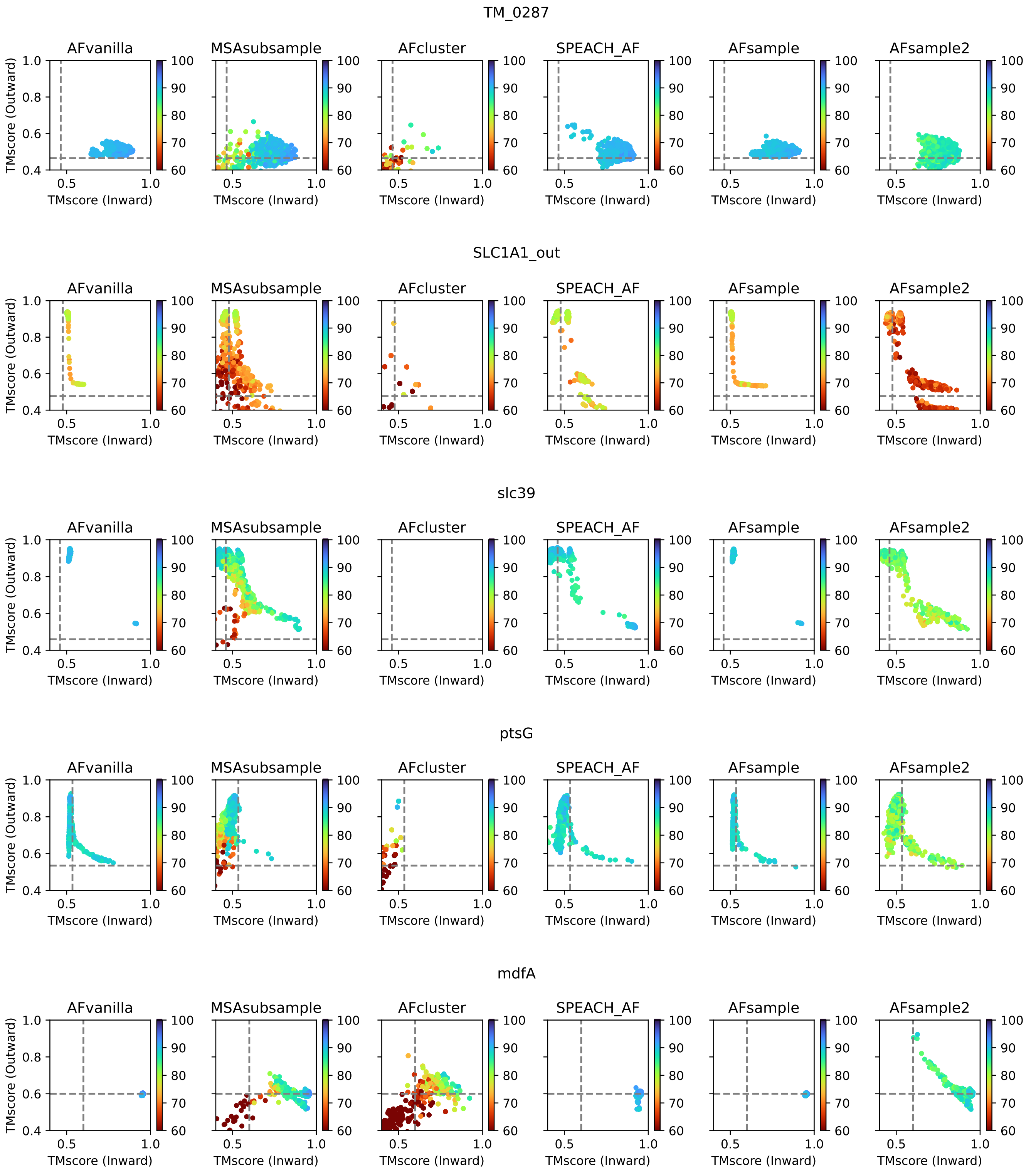

Fig. S7. Diversity plots to capture the similarity of model ensembles with reference states for all targets in the transporter dataset (continued on next page)

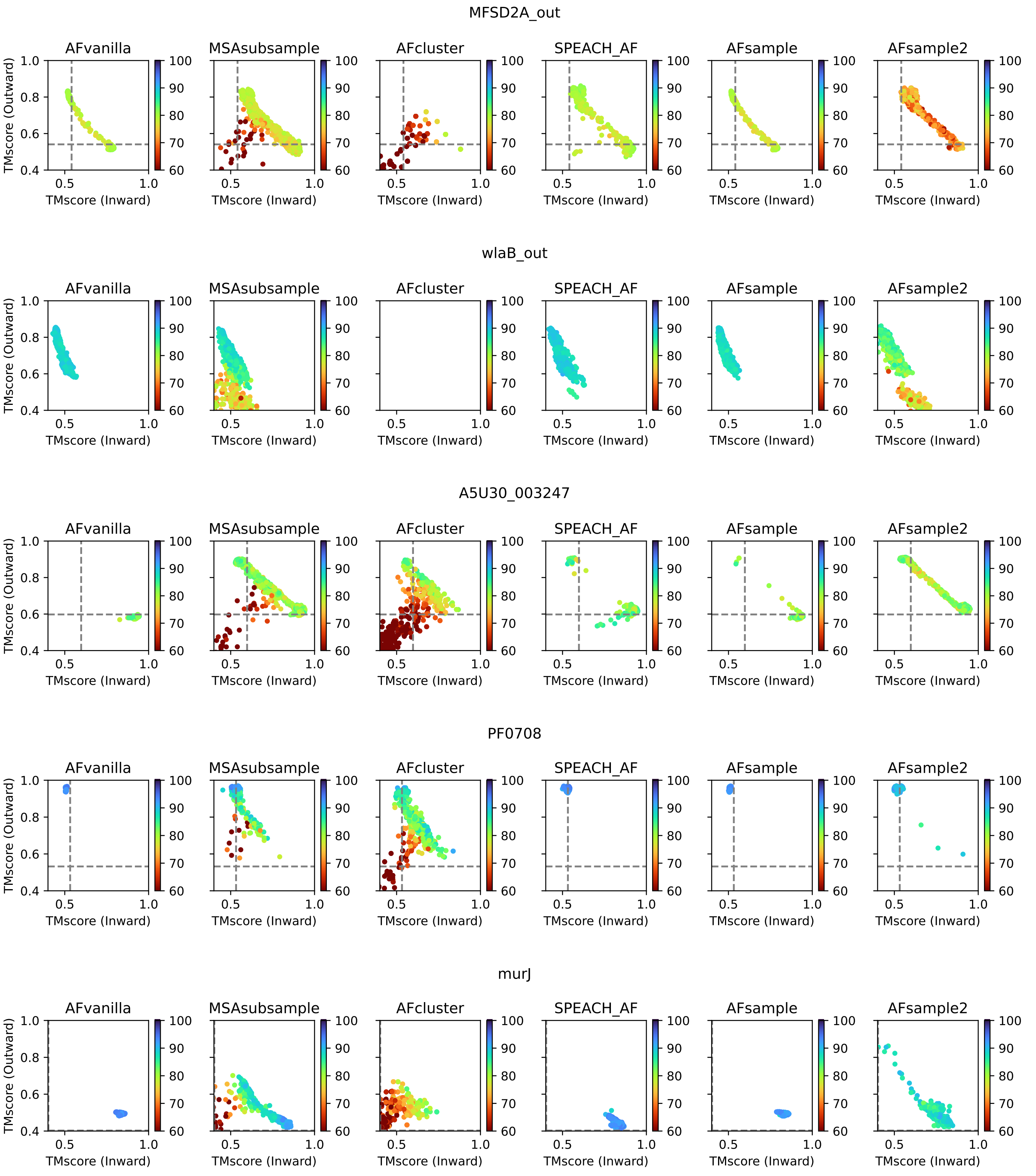

Fig. S7. Diversity plots to capture the similarity of model ensembles with reference states for all targets in the transporter dataset (continued on next page)

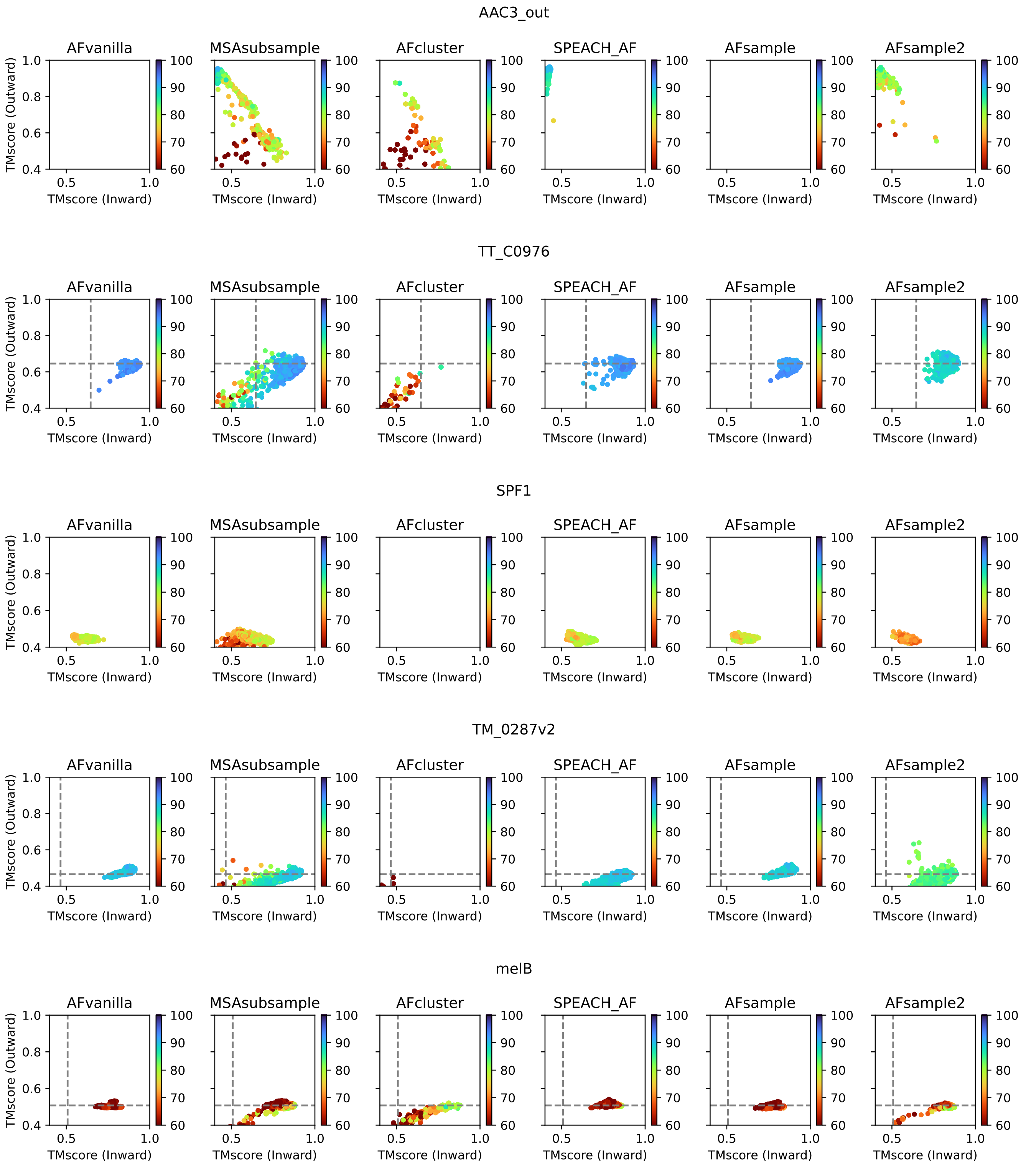

Fig. S7. Diversity plots to capture the similarity of model ensembles with reference states for all targets in the transporter dataset (continued on next page)

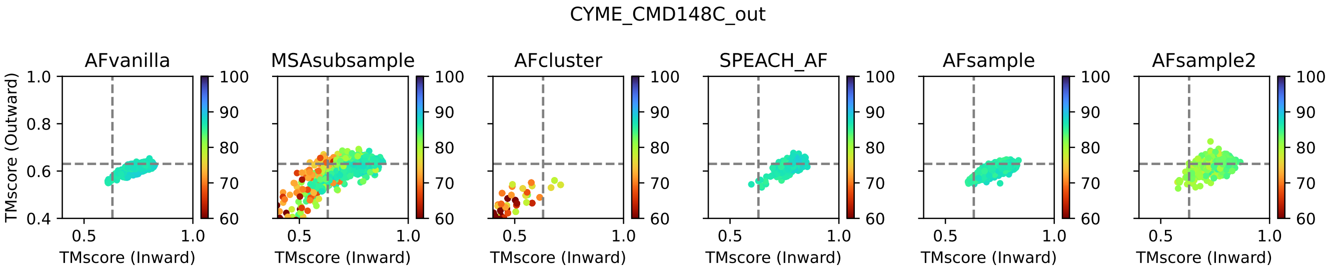

Fig. S7. Diversity plots to capture the similarity of model ensembles with reference states for all targets in the transporter dataset

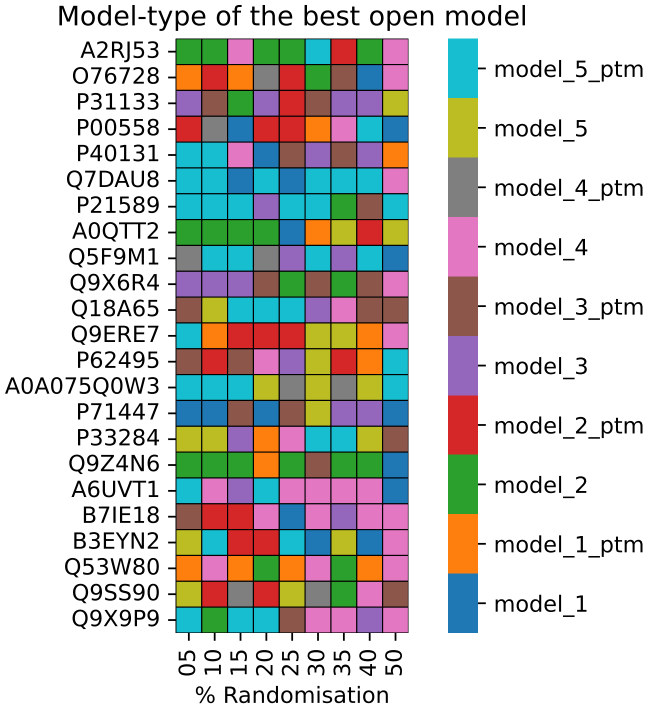

(a) Per-protein best TM-score at each level of MSA-randomization.

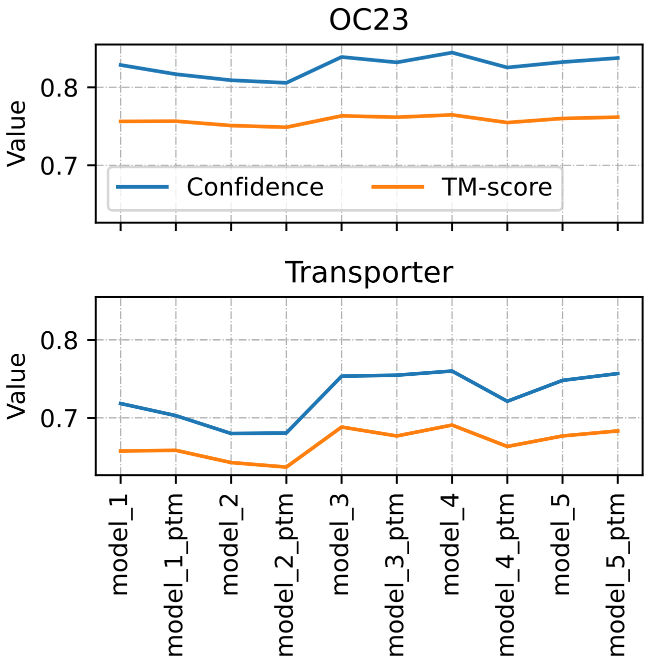

(b) Distribution of mean confidence scores in model ensembles for various model types.

Fig. S8. (a) Analysis to see if any of the model-types are favoured to generate the best-open model. Since the heatmap does not show any clear pattern, it can be inferred that model-type preferences is completely dependent on individual protein.

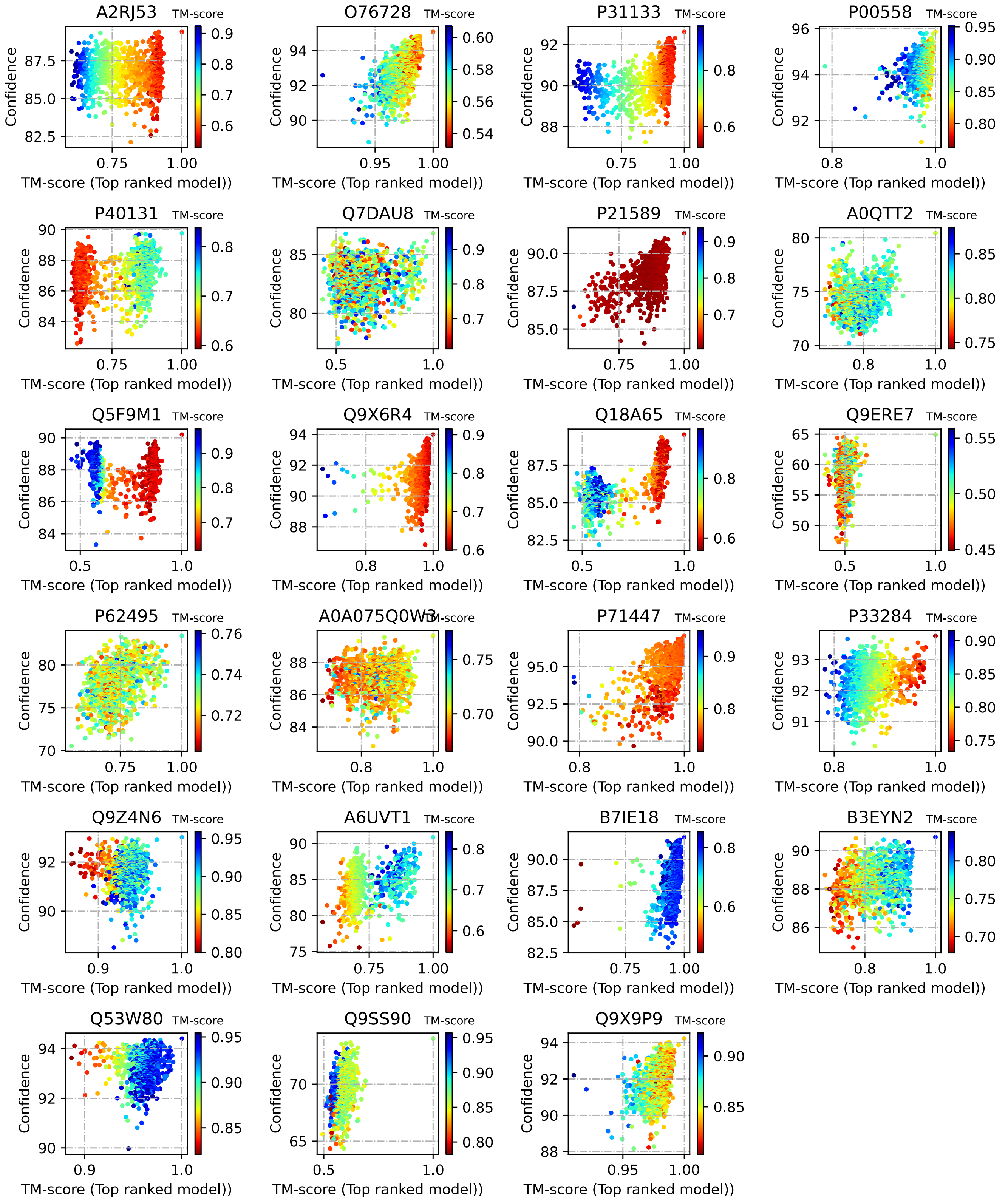

Fig. S9. Reference-free state determination. The assumption regarding alternate state to be far from the most confident model holds for the OC23 dataset. The color-gradient denotes closeness to the alternate (open) state.

Uniprotid	pdbid_open	pdbid_closed	Length	TM-score	RMSD
A2RJ53	3fto_A	3drf_A	576	0.507	4.660
O76728	4bp8_A	4bp9_A	714	0.523	4.310
P31133	6yed_A	6ye0_A	343	0.565	3.790
P00558	2xe6_A	2wzd_A	416	0.598	3.620
P40131	3tee_A	3vjp_A	197	0.612	2.480
Q7DAU8	3l6g_A	3l6h_A	253	0.617	3.340
P21589	7qga_A	4h2i_A	522	0.621	4.270
A0QTT2	7cy2_A	7cyr_A	438	0.633	2.800
Q5F9M1	3zsf_A	2yln_A	258	0.636	3.450
Q9X6R4	3iuj_A	3iuq_A	689	0.648	3.330
Q18A65	6hnj_A	6hni_A	316	0.656	3.010
Q9ERE7	2rqm_A	2rqk_A	139	0.664	1.600
P62495	2ktv_A	2ktu_A	161	0.708	3.120
A0A075Q0W3	6mka_A	6mkj_A	639	0.720	3.460
P71447	2wfa_A	2wf5_A	220	0.748	2.920
P33284	3o6w_A	3o8m_A	484	0.749	3.140
Q9Z4N6	1si1_A	1si0_A	319	0.749	2.400
A6UVT1	6hac_A	6hae_A	341	0.754	2.880
B7IE18	6nc7_A	6nc6_A	474	0.755	2.790
B3EYN2	5ho2_A	5ho0_A	847	0.766	3.450
Q53W80	7c63_A	7c66_A	415	0.781	2.040
Q9SS90	6k8b_A	6k85_B	290	0.824	2.180
Q9X9P9	2olo_A	2oln_A	388	0.837	1.760

Table S1. Summary of targets in OC23 dataset.

Uniprotid	pdbid_open	pdbid_closed	Length	TM-score	RMSD
Q82GL5	7cl7_A	7cl8_A	399	0.85	1.85
P61316	2zpd_A	2zpc_A	181	0.85	1.91
P0A4G2	4uto_A	4utp_A	308	0.86	1.61
Q9UBV7	4irp_A	4irq_A	246	0.86	1.29
P76045	2iwv_A	2iww_A	279	0.88	2.18
P48635	2wio_A	2jjn_A	396	0.89	1.94
Q8A5V9	3quq_A	3qu2_A	243	0.89	1.27
A0R629	5eqd_B	5eqd_A	399	0.90	1.68
P18031	5k9v_A	5k9w_A	300	0.90	0.95
A0A1J6PWI8	6jmx_A	6jmy_A	253	0.91	1.67
P29350	4hjp_A	4hjq_A	285	0.91	1.60
P0CG48	3ns8_A	2n2k_A	75	0.92	1.26
P9WNX1	4klx_A	6nni_A	158	0.92	1.08
P15291	2fy7_A	2fyc_B	272	0.92	1.29
C7C425	6xcs_A	6xcq_A	417	0.94	1.15
P14902	7p0n_A	7nge_A	388	0.95	0.33
O34926	3nc3_A	3nc3_B	404	0.95	1.16
Q72HW2	5afa_A	6tyr_A	438	0.97	0.99
P18965	3s9b_A	3s9a_A	235	0.99	0.44
Q9U6Y3	6qsm_A	6qsl_A	216	0.99	0.40
J9UN47	4psp_A	4ni3_A	584	0.99	0.47
P00918	3m1q_A	3m1w_A	259	1.00	0.29

Table S2. Summary of targets in OC>85 dataset.

Target	pdbid (IF)	pdbid (OF)	Length	TM-score	RMSD
TM_0287	3QF4_A	6QV1_C	587	0.465	5.160
SLC1A1_OUT	6S3Q_A	6X3E_A	526	0.477	4.920
SLC39	6BTX_A	5AYM_A	440	0.460	5.310
PTSG	6BVG_A	5IWS_A	470	0.535	4.840
MDFA	6VS1_A	6GV1_A	410	0.600	4.200
MFSD2A_OUT	7MJS_X	7N98_A	534	0.541	4.540
WLAB_OUT	5C78_A	6HRC_D	564	0.385	4.990
A5U30_003247	6E9N_B	6E9O_B	460	0.598	4.180
PF0708	6FHZ_A	3VVS_A	461	0.532	4.730
MURJ	6NC7_A	6NC9_A	475	0.403	5.600
AAC3_OUT	6GCI_A	4C9J_B	322	0.379	4.820
TT_C0976	5MKK_B	6RAJ_B	578	0.645	4.450
SPF1	6XMS_A	6XMU_A	1239	0.391	0.874
MELB	7L17_A	4M64_A	486	0.508	4.400
CYME_CMD148C_OUT	6A6N_A	7DQV_A	620	0.630	4.300
TM_0287V2	4Q4A_B	6QV1_B	599	0.465	5.420

Table S3. Summary of targets in transporter dataset. (IF: Inward facing, OF: Outward facing)
